# Doses in organs at risk during head and neck radiotherapy using IMRT and 3D-CRT

**DOI:** 10.2478/v10019-012-0050-y

**Published:** 2012-11-09

**Authors:** Magdalena Peszynska-Piorun, Julian Malicki, Wojciech Golusinski

**Affiliations:** 1 Medical Physics Department, Mikolaj Kopernik Memorial Regional Specialized Hospital, Lodz, Poland; 2 Medical Physics Department, Greater Poland Cancer Centre, Poznan, Poland; 3 Electroradiology Department, University of Medical Sciences, Poznan, Poland; 4 Medical Physics Department, Adam Mickiewicz University, Poznan, Poland; 5 Head and Neck Surgery Department, Greater Poland Cancer Centre, Poznan, Poland; 6 Maxillofacial Surgery Department, University of Medical Sciences, Poznan, Poland

**Keywords:** radiotherapy, IMRT, 3D-CRT, head and neck cancer

## Abstract

**Background.:**

Treatment planning for head and neck (H&N) cancer is complex due to the number of organs at risk (OAR) located near the planning treatment volume (PTV). Distant OAR must also be taken into consideration. Intensity-modulated radiotherapy (IMRT) and three-dimensional conformal radiotherapy (3D-CRT) are both common H&N treatment techniques with very different planning approaches. Although IMRT allows a better dose conformity in PTV, there is much less evidence as to which technique less dose to OAR is delivered. Therefore, the aim of the study was to compare IMRT to 3D-CRT treatment in terms of dose distribution to OAR in H&N cancer.

**Patients and methods.:**

This was a prospective study of a series of 25 patients diagnosed with stage cT_3–4_N_0–2_ laryngeal cancer. All patients underwent total laryngectomy and bilateral selective neck dissections. In all cases, patients were treated with IMRT, although a 3D-CRT treatment plan was also developed for the comparative analysis. To compare doses to specific OAR, we developed a new comparative index based on sub-volumes.

**Results.:**

In general, IMRT appears to deliver comparable or greater doses to OAR, although the only significant differences were found in the cerebellum, in which 3D-CRT was found to better spare the organ.

**Conclusions.:**

Organs located outside of the IMRT beam (*i.e.*, distant organs) are generally thought to be well-spared. However, the results of this study show that, in the case of the cerebellum, this was not true. This finding suggests that larger studies should be performed to understand the effects of IMRT on distant tissues. Anthropomorphic phantom studies could also confirm these results.

## Introduction

The global incidence of head and neck cancer is about 650000 cases per year, which represents about 6% cancer incidence (skin cancer excluded). Radiotherapy plays an important role in the treatment of head & neck (H&N) cancers.^1^,^2^ However, patients, who undergo irradiation, require a comprehensive pre-treatment evaluation.^3^ In recent decades, the treatment for H&N cancer has moved from two-dimensional radiotherapy to three-dimensional conformal radiotherapy (3D-CRT) and recently also to intensity-modulated radiotherapy (IMRT). Both 3D-CRT and IMRT represent a significant advance over the conventional radiotherapy because they increase dose delivery accuracy while sparing surrounding normal tissues and organs at risk (OAR). The dose-modulating ability of IMRT gives a theoretical advantage over 3D-CRT, which recently has been also supported in the clinical trial.^4^,^5^

IMRT is still a relatively new technique and although its superiority over 3D-CRT in term of tumor dose coverage is clear, there is still some concerns about doses in OAR.^5^–^8^ Nevertheless, IMRT has become the primary treatment technique in H&N cancers due to its better dose conformity, demonstrated very well in the treatment of lesions with complex anatomy which are adjacent to vital structures such as the spinal cord or brain stem.^7^,^9^–^11^ However, this improved the conformity to the target which may have an undesirable drawback as the large number of fields used in IMRT can potentially lead to higher doses outside the planning treatment volume (PTV).^9^,^12^,^13^ IMRT also requires more complex quality assurance procedures.^2^,^14^–^17^

Few studies have compared IMRT to 3D-CRT in H&N radiotherapy to evaluate doses to OARs located outside of the PTV.^18^,^19^ The present study was aimed to compare differences in calculated dose distributions to OAR for IMRT *vs.* 3D-CRT in a series of 25 patients with H&N cancer. In the article we focused on organs that had not been previously analyzed *i.e.*: thyroid gland, mandible, brain stem, brain and the cerebellum.

## Patients and methods

This was a prospective study of a series of 25 patients diagnosed with stage cT_3–4_N_0–2_ laryngeal cancer. All patients underwent total laryngectomy and bilateral selective neck dissections. In all cases, patients were treated with IMRT, although a 3D-CRT treatment plan was also developed for the comparative analysis.

The IMRT treatment plan had prescription of 54 Gy delivered in fractions of 1.8 Gy (30 fractions) for the PTV1 with a simultaneous integrated boost (SIB) of 60 Gy at 2 Gy/fraction to the PTV2. A set of 6 non-coplanar 6 MV photon beams was used for 15 patients that entered the study first and a set of 7 non-coplanar 6 MV photon beams for 10 patients who followed the first group. Originally, 6-field IMRT plan was considered as a standard setup. Afterwards, as a result of a thorough comparison of the 7-field and the 6-field technique, the latter one was used. The gantry angles used for the 6-field technique were as follows: 35° (collimator 0°, table 0°), 110° (collimator 10°, table 15°), 180° (collimator 0°, 0°), 250° (collimator 350°, table 345°), 325° (collimator 0°, table 0°), 340° (collimator 0°, table 0°). Respectively, for the 7-field technique gantry angles were: 0° (collimator 0°, table 0°), 40° (collimator 0°, table 0°), 110° (collimator 108.9°, table 10°), 150° (collimator 5°, table 0°), 210° (collimator 352.7°, table 0°), 250° (collimator 265°, table 350°), 320° (collimator 90°, table 0°).

The 3D-CRT treatment plans included two separate phases, one for the primary treatment and the second plan for the boost. Fifty Gy (2 Gy/fraction) were prescribed to the PTV1. The second phase was a boost of 10 Gy at 2 Gy/fraction to the PTV2, for a total of 30 fractions (the same as for IMRT). Both plans were mainly based on 6 MV photons, except for fields with small weights (only in phase 1), in which case 15 MV photons were used. The first phase called for 10 to 12 fields with extra 2–3 fields was used for the boost phase. All fields were coplanar, in some cases opposed: (90° and 270°, 0° and 180° but also 60°, 300°, 100°, 260°). In 2–4 fields the collimator angle was ± 18° (first phase) and ± 5° (second phase). Many field wedges (mostly 45°) had to be used due to contouring irregularity inherent in H&N treatments. In some cases, the irradiated regions could not be completely covered by a homogenous dose and wedged fields, so half-beams were also used.

Treatment plans for both techniques were calculated and optimized using the Eclipse Treatment Planning System (Varian, Medical System, Palo Alto, CA, USA) with AAA algorithm. The IMRT used a dynamic sliding window technique. The CT images acquired included the whole head and the neck to the level of T_3_–T_4_ (thoracic vertebra). The clinical target volume (CTV), PTV1, PTV2/boost and OAR volumes were delineated by the same radiation oncologist for all patients and techniques. The following OARs were chosen for the dose comparison: thyroid gland, mandible, brain stem, cerebellum, brain. For 3D-CRT doses in all structures were optimized manually while for IMRT the automated optimization was performed for the following structures: PTVs, CTVs, salivary glands, spinal cord and the area above the PTV, which often overlapped with the mandible and the back side of the head/occipital bone and the neck at shallow depth under the skin, to spare the hair.

Treatment planning was performed in accordance with the International Commission on Radiation Units and Measurements (ICRU) reports on H&N cancer and IMRT (#50, #62 and #83)^10^,^14^,^20^–^23^, which recommend a homogenous dose distribution (range, 95% – 107% of the prescribed dose) to the PTV. Values for the constraints used in the process of dose optimization have been as follows: dose fractionation 1.8–2.0 Gy. Following: Brain, D(33%) ≤60 Gy, D(66%)≤50 Gy, D(100%)≤45 Gy with priority from 40 to 80; Brain stem, Dmax=54 Gy with priority circa 80, Spinal cord, Dmax=48 Gy with priority circa 110, Salivary gland, Dmean≤30 Gy, Mandible which is a part of area above the PTV, Dmax≤70 Gy with priority circa 90.^24^,^25^

For particular patients, additional constraints have been used, *i.e.* for salivary glands D(70%)≤5 Gy, D(50%)≤18 Gy, D(30%)≤25 Gy, D(15%)≤ 35Gy with priority from 50 to 90. Function “normal tissue objective” with priority 100 was used to the further lower dose in all OARs. The modification of constraints and priorities is a part of treatment planning. It makes the optimization process slightly subjective which, however, is inevitable as a patient anatomy differs from one to another, and parameters have to be modified in order to obtain the uniform dose in PTV and the low dose in the optimized OARs

Standard parameters produced by the treatment planning software (TPS) were not sufficient to an effectively and quantitatively assess the differences between IMRT and 3D-CRT plans. Doses were very inhomogeneous and often low in OARs, making it difficult to identify small deviations. To more precisely evaluate a dose distribution to the OARs, we realized that we needed more detailed information aside from the standard maximum, minimum, and mean values. Doses at 0% volume, 30% volume, 60% volume, 90% volume (notation D(0%), D(30%), D(60%), D(90%)) are considered to be representative in normal tissue complication probability (NTCP) models.^21^,^26^ For this reason, we evaluated doses for each OAR in 4 sub-volumes (0%, 30%, 60%, 90% of the relative volume) and wherever more complex information was desired we evaluated doses in 10 sub-volumes (with each sub-volume representing from 0% to 90% of the volume, with a step of 10% of a relative volume). For each of the sub-volumes, maximal doses were read from the cumulative histogram for both IMRT and 3D-CRT, and then the differences and mean values for all sub-volumes were calculated.

In this paper, we present all 10 sub-volume doses in the cerebellum and brain. For other organs evaluated, we present doses only for 4 sub-volumes (90%, 60%, 30%, 0%).

### Statistical analysis and ethical consideration

After calculating the dose received by the various sub-volumes according to the treatment technique (IMRT or 3D-CRT), we used the Shapiro-Wilk test to assess the distribution of the data, which was found to be non-Gaussian. Therefore, the nonparametric U Mann-Whitney rank sum test was applied, with a significance level of 0.05. We used the software programs Statistica 8.0 and Origin 8.0 (OriginLab, Northampton) to perform the statistical analysis.

The prospective study was carried out according to the Declaration of Helsinki.

## Results

**FIGURE 1:**
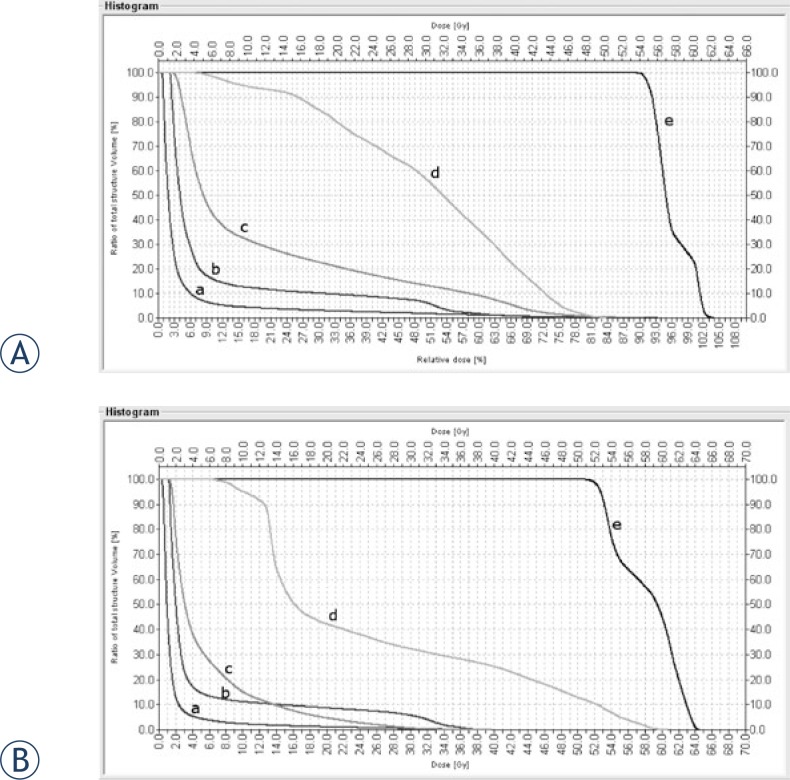
Dose volume histogram for the IMRT (A) and 3D-CRT (B) plans. The organs at risk are: cerebellum (c), brain stem (b), mandible (d), thyroid gland (e) and brain (a).

As discussed above, the primary analysis for this study was based on an index of sub-volumes for the OARs. However, prior to evaluating these sub-volumes, we calculated the dose-volume histogram (DVH) for all 25 patients and OARs evaluated in this study. The histograms for both IMRT and 3D-CRT presented similar trends for the dose-volume dependence of the OAR, although it was not possible to detect significant differences. As a result, we concluded that conventional histograms were not sufficient to perform a dose distribution comparison. The histogram of a patient in Figure 1 was taken to demonstrate the similarity between the two techniques.

Table 1 shows the doses received by the OARs for all patients. The only positive values are found for the cerebellum while the other OAR’ show a range of positive and negative values (−10 Gy to +14.5 Gy), with less dispersion for the brain stem. The brain shows a consistent trend with minimal deviation between techniques, with many values near 0.

**FIGURE 2:**
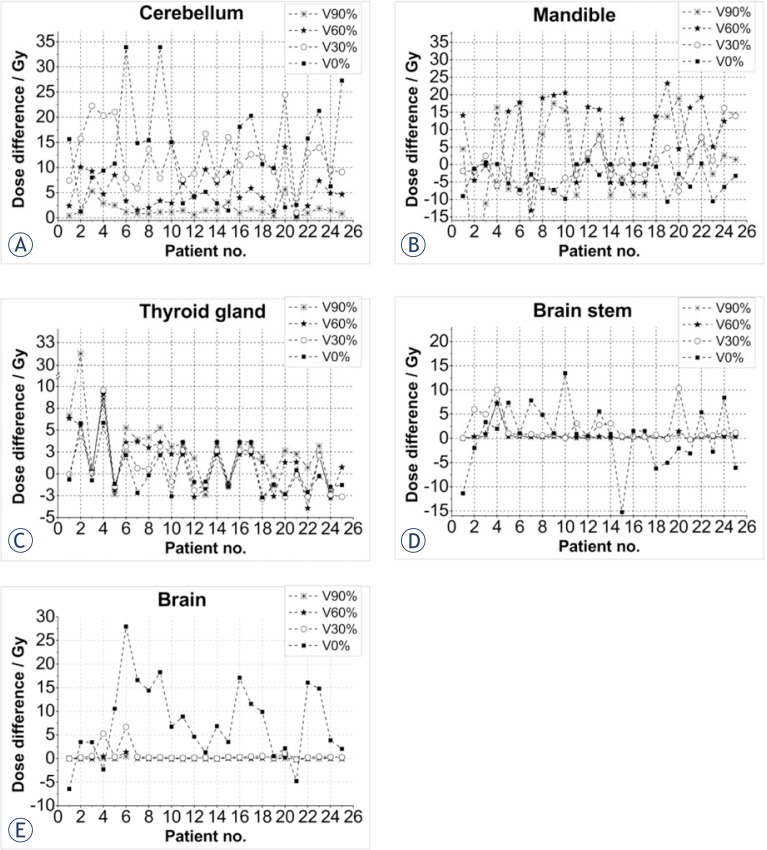
Dose differences (IMRT dose - 3D-CRT dose) for the organs at risk: cerebellum (A), mandible (B), thyroid gland (C), brain stem (D) and brain (E).

Dose differences between the two treatment techniques can be more clearly seen in the sub-volumes. In Figure 2, dose plots for chosen OARs are shown for all 25 patients. Dose differences (IMRT_dose_ - 3D-CRT_dose_) for cerebellum (a), mandible (b), thyroid gland (c), brain stem (d) and brain (e) are shown.

**FIGURE 3:**
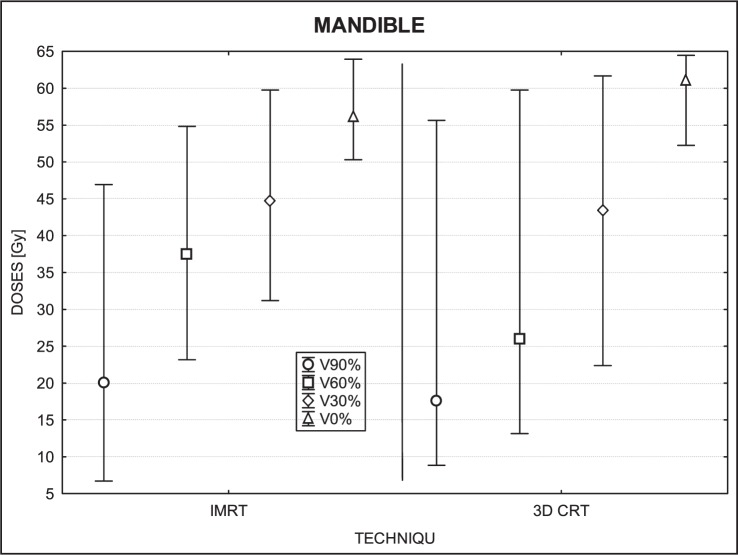
A box plot of the mandible for 4 volumes: V90%, V60%, V30% and V0%. Middle point is the mean value; the box, standard deviation; and whisker, Min – Max value.

**FIGURE 4:**
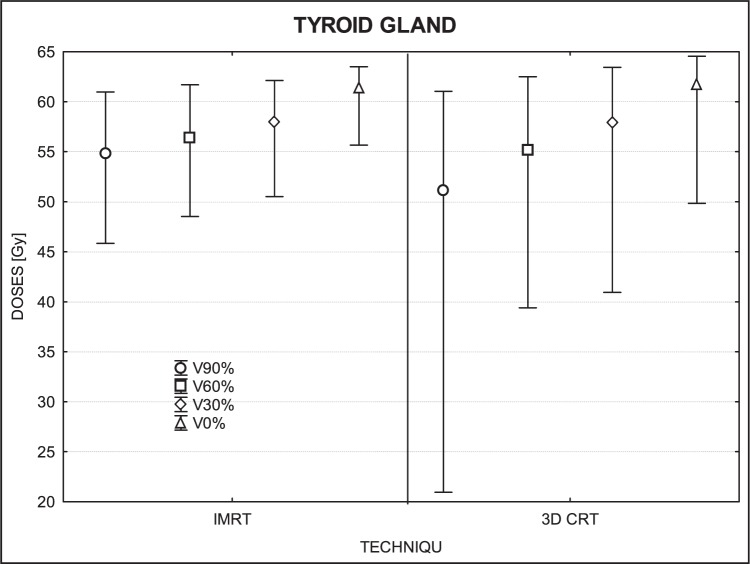
A box plot of thyroid gland for 4 volumes: V90%, V60%, V30% and V0%. Middle point is the mean value; the box, standard deviation; and whisker, Min – Max value.

**FIGURE 5:**
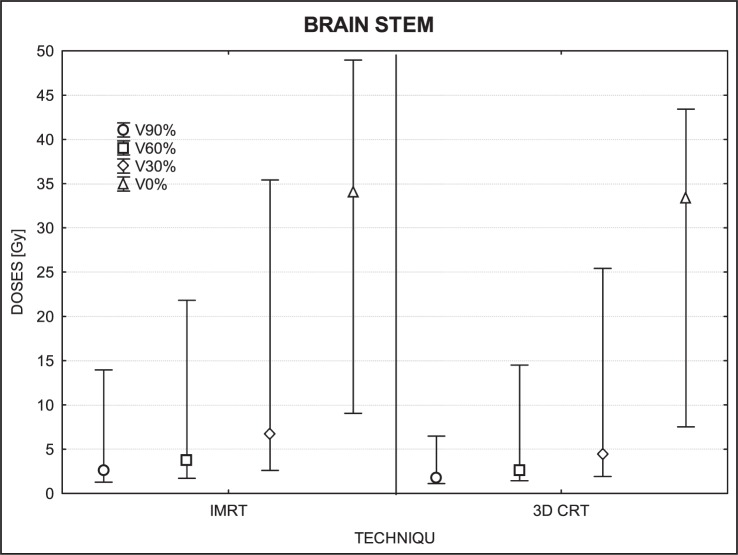
A box plot of brain stem for 4 volumes: V90%, V60%, V30% and V0%. Middle point is the mean value; the box, standard deviation; and whisker, Min – Max value.

**FIGURE 6:**
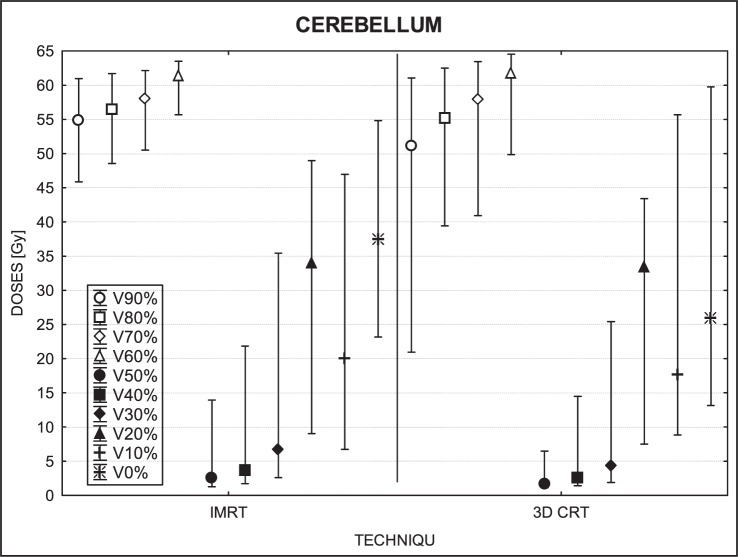
A box plot of cerebellum for 10 volumes: from V90% to V0%. Middle point is the mean value; the box, standard deviation; and whisker, Min – Max value.

**FIGURE 7:**
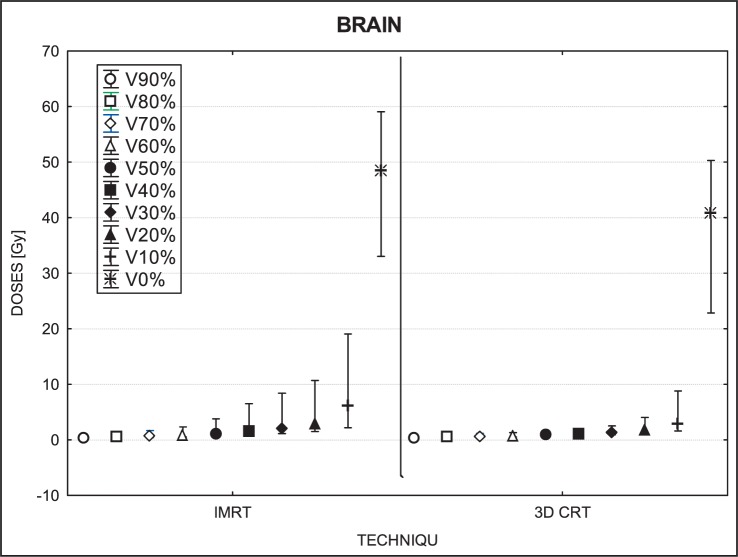
A box plot of brain for 10 volumes: from V90% to V0%. Middle point is the mean value; the box, standard deviation; and whisker, Min – Max value.

Figures 3 to 7 show the mean dose, maximal and minimal doses, and standard deviation in sub-volumes for the study group, for each OAR at IMRT and 3D-CRT.

Table 2 shows the mean radiation doses to the cerebellum for the group (25 patients) by the treatment technique (IMRT, 3D-CRT). The differential dose is also shown.

IMRT = intensity-modulated radiotherapy; 3D-CRT = three-dimensional conformal radiotherapy

The statistical analysis for the mandible showed significant differences in doses for 3 sub-volumes (90%, 60% and 0%; P= 0.04, 0.00 and 0.001, respectively). The upper-tailed Mann Whitney test showed that doses in sub-volume of 90% (p=0.02) and sub-volume of 60% (p=0.0006) were greater for IMRT than for 3D-CRT. The lower-tailed Mann Whitney test showed that maximal doses (which correspond to the dose at 0% of the volume) were lower for IMRT (p=0.00003) than for 3D-CRT. The only sub-volume for which no significant difference was detected was for 30% (p= 0.91) (Figure 3).

In the thyroid gland, we found significant differences for 90% of volume (p= 0.01) and 0% (p=0.01), but no significant differences in sub-volume of 60% (p=0.09) or 30% (p=0.66). At the p-level of 0.005 for the upper-tailed test, the IMRT doses in sub-volume of 90% were significantly higher than those of 3D-CRT while the lower-tailed test revealed that maximum doses were lower with IMRT than 3D-CRT (Figure 4).

The dose differences in the brain stem were not statistically significant (90% of volume, p=0.06; 30%, p=0.07; 0%, p=0.55) except for 60% of volume (p=0.034) (Figure 5).

For low dose areas of the brain (*i.e*., sub-volumes ranging from 40% to 90%), no significant differences were found (40%, p=0.11; 50%, p=0.40; 60%, p=0.85; 70%, p=0.60; 80%, p=0.35; 90%, p=0.94). The only significant differences found were in the smaller volumes (0% to 30%) that received higher doses overall. For these sub-volumes, IMRT delivered higher doses than 3D-CTR, with a mean difference of 5–8 Gy. For 0% of volume (IMRT- 48.76 Gy; 3D-CRT- 41.20 Gy), p=0.0001; for 10% (IMRT- 6.18 Gy; 3D-CRT- 2.99 Gy), p=0.00; for 20% (IMRT- 2.96 Gy; 3D-CRT-1.82 Gy), p=0.0005; for 30% (IMRT-2.09 Gy; 3D-CRT- 1.38 Gy), p=0.01. The IMRT techniques delivered higher doses to these volumes, with a higher maximum dose (30%, p=0.003; 20%, p=0.0003; 10%, p=0.0005; 0%, p=0.0001).

## Discussion

Of all the organs at risk evaluated, the cerebellum was the only OAR in our study with a positive mean dose differential (D_IMRT_ – D_3D-CRT_), meaning that 3D-CRT delivered lower doses to the cerebellum than IMRT. Moreover, we found that the bigger the volume measured the smaller and more constant the differential dose was.

### Brain

Dose distributions in the whole brain, including the cerebellum, were dependent on the volume evaluated. For volumes ranging from 40% to 90%, IMRT doses were lower than 3D-CRT while volumes from 0% to 30% the 3D-CRT doses were lower.

### Mandible and thyroid

No consistent trends were observed for the mandible and thyroid gland. For some patients, IMRT doses were higher than 3D-CRT while for others it was exactly the reverse. However, it is interesting to note that the maximum dose for the mandible was, in most cases, lower for IMRT. Much bigger deviations in doses were observed in the volume of 60% and 90%, because the differences in low dose regions were larger. IMRT was superior to 3D-CRT in sparing the mandible in 6 patients while the reverse was true in 3 patients. Similarly, doses to the thyroid gland were lower (better sparing) with IMRT for 5 patients and by 3D-CRT for 8 patients.

### Brain stem

No significant differences were observed in the brain stem as the dose distributions in this organ were similar for both techniques. The detected differences were very small and could have been due to chance. The maximum dose varied in a few patients, but this could be caused by the small volume of this organ leading to a point dose, pixel or voxel, in which the maximum dose was found.

### Cerebellum

Relative to the PTV, the cerebellum was considered a distant organ and unlike the other organs, IMRT resulted in significantly higher doses to the cerebellum in all sub-volumes. The reason for this difference is evident: not all of the 3D-CRT treatment fields covered the cerebellum whereas in IMRT, a greater number of fields passed through the cerebellum. So, even though the IMRT doses were lower, the overall accumulated dose was greater with IMRT. These differences were especially notable in small sub-volumes. In general, we found that 3D-CRT produced more homogenous doses to the brain (Figures 2A, 6) than IMRT, even though the mean dose of 3D-CRT was higher.

Our results are interesting in that they show that both techniques are largely similar in terms of OAR irradiation, with the only notable exception being the cerebellum, which receives more radiation with IMRT than 3D-CRT. These results do not contradict advantages of IMRT-that because dose distribution is more conformal than 3D-CRT, it is also less toxic to adjacent healthy tissues. However, we should keep in mind that data and follow up for IMRT are still relatively limited.^7^,^10^,^27^

A review of the literature shows varying data, with in principle IMRT superior for some OARs, however the concern of potentially higher doses in distant organs is noted.^4^,^5^,^7^,^14^,^18^,^28^ In this work, we confirmed that IMRT allowed on better reduction of doses in OARs however in particular situation the 3D-CRT may allow on better spearing the cerebellum, thus the attention is required in such situation.

A recent study by Chen *et al.* compared dose-volume characteristics of the brachial plexus for IMRT and CRT and found out that a dose to the brachial plexus was significantly increased among patients undergoing IMRT compared with CRT.^28^ However, a different study comparing 3D-CRT and IMRT for thyroid cancer found out that the dose to the spinal cord was 12 Gy less with IMRT and the coverage of the target volume was also better, with a smaller standard deviation (4.65% for 3D-CRT *vs*. 1.81% for IMRT). Longobardi *et al*. found out that IMRT dose planed provide more uniform coverage of the PTV than 3D-CRT and IMRT resulted in a significant reduction of mean and/or maximum doses to OAR; of particular note, these authors reported that the mean dose to the parotid glands decreased by a mean of 13.5 Gy with the use of IMRT vs. 3D-CRT.^29^ The revealed differences in dose parameters between evaluated techniques were small due to the improvement in optimization applied in all radiotherapy techniques, thus detection tools and methods that allowed to trace the differences had to be appropriately accurate.^12^,^30^,^31^

Both techniques present advantages and disadvantages. Using the 3D CRT does not allow for simultaneously integrated boost and in consequence two separate courses of radiotherapy are necessary, with the risk of the field overlap that may cause hot spots and some inhomogeneity in dose distributions. These drawbacks are not insignificant and may, therefore, limit the number of indications for 3D-CRT.^5^,^32^,^33^

IMRT, in contrast, although more complex^2^,^13^,^16^,^34^, allows for more homogenous dose deposition in the target while sparing surrounding normal tissues. Moreover, IMRT allows for integration of the boost dose (SIB) in one course of the treatment, thus resulting in a higher fraction dose (hypofractionation, *i.e.* dose fraction greater than 2 Gy) while the PTV receives a dose from range of 1.8 Gy to 2.0 Gy at the same time.

Both 3D-CRT and IMRT have their advantages, and should be selected on a case by case basis. Although IMRT is technically more sophisticated, 3D-CRT is perfectly capable of delivering the appropriate dose to the target.^1^,^5^,^14^,^35^ However, the primary disadvantage with 3D-CRT is a dose distribution, which is often uneven. The main benefit of IMRT over 3D-CRT is the ability to optimize the treatment in the planning stage to deliver the appropriate dose to the target while optimizing the plan to adhere to the OAR constraints. Particularly, the IMRT is useful in the regions where the tumor is located close to OARs. The OARs, which lay outside but very near the PTV may be better spared, when using IMRT than 3D-CRT technique. In fact, this is why most experts prefer IMRT for H&N cancers. The main drawback of IMRT seems to be that the dose is not optimized for distant OARs, *i.e.*, those that are not located in the path of the beam and, thus, not considered crucial during planning.^36^

## Conclusions

This study has shown the feasibility of using an index of sub-volumes to better evaluate a dose distribution to OARs located outside of the PTV. We believe that this new approach allows for an effective dose comparison of the dose distributions at locations with a steep dose gradient.

The findings of our study show that IMRT allowed the better reduction of doses in OARs, however, in particular situations the 3D-CRT may allow better sparing the cerebellum and this is an important factor to consider when planning treatments in H&N cancers. Particularly, the OAR for which dose was not optimized, might receive a higher dose (jello effect). It could occur because during IMRT larger body part is usually irradiated with a small dose and not all OARs can be taken into an optimization process.

## Figures and Tables

**TABLE 1:** The authors’ index of the mean dose difference (D_IMRT_ – D_3DCRT_) for the organs at risk and sub-volumes evaluated for this series of 25 patients. The index value was the mean dose difference (D_IMRT_ – D_3DCRT_) for the doses in 10 sub-volumes for cerebellum and brain and of 4 sub-volumes for thyroid gland, brain stem, and mandible. A positive value indicated that the mean dose for intensity-modulated radiotherapy (IMRT) was greater than the mean three-dimensional conformal radiotherapy (3D-CRT) dose.

**Patient Number**	**Cerebellum Dose [Gy]**	**Thyroid gland Dose [Gy]**	**Brain stem Dose [Gy]**	**Mandible Dose [Gy]**	**Brain Dose [Gy]**
**1**	5.75	3.09	−2.82	1.92	−0.66
**2**	9.82	11.83	1.15	−9.68	0.70
**3**	13.13	0.01	2.43	−2.06	1.16
**4**	12.08	8.28	6.69	1.04	2.45
**5**	12.81	−1.70	2.28	0.32	1.80
**6**	8.58	3.53	0.67	5.03	6.59
**7**	6.43	1.51	2.30	−10.11	1.92
**8**	9.06	1.88	1.49	4.09	1.59
**9**	8.58	3.53	0.67	5.53	2.08
**10**	6.74	0.48	3.49	5.59	0.74
**11**	7.25	2.49	−3.83	1.52	1.03
**12**	5.54	−0.93	0.27	5.65	0.71
**13**	10.05	−1.55	2.22	7.13	0.39
**14**	5.02	2.98	1.03	−4.18	0.86
**15**	8.89	−1.26	−3.64	1.22	0.73
**16**	8.85	0.75	0.59	0.93	2.05
**17**	10.72	1.83	−1.83	11.97	1.57
**18**	8.53	−0.57	−1.20	7.09	1.57
**19**	5.43	−1.34	−1.22	7.74	0.20
**20**	14.50	−0.25	2.59	3.26	1.70
**21**	1.60	0.98	−0.97	3.28	−0.65
**22**	8.84	−1.98	1.66	8.66	1.74
**23**	11.61	1.79	−0.39	−1.71	2.20
**24**	6.91	−2.17	2.57	6.17	0.88
**25**	8.33	2.16	−1.07	6.54	0.54

**TABLE 2:** Mean radiation doses (Gy) to the cerebellum for the group (25 patients) by the treatment technique (IMRT, 3D-CRT). The differential dose for all sub-volumes (from 0% to 90%, step of 10%) is also shown.

**Sub-volume**	**Mean dose (IMRT)**	**Mean dose (3D-CRT)**	**Dose differential (IMRT – 3D-CRT)**
90% of V	4.10	2.13	1.98
80% of V	5.49	2.50	2.99
70% of V	7.11	2.87	4.24
60% of V	9.18	3.33	5.85
50% of V	11.86	3.98	7.88
40% of V	15.35	4.87	10.48
30% of V	19.57	6.37	13.21
20% of V	24.77	9.23	15.50
10% of V	31.49	15.10	16.39
0% of V	47.50	34.91	12.59
